# Natural compounds targeting mitochondrial dysfunction: emerging therapeutics for target organ damage in hypertension

**DOI:** 10.3389/fphar.2023.1209890

**Published:** 2023-06-15

**Authors:** Xiaolin Liao, Yuanshan Han, Ying He, Jianjun Liu, Yuhong Wang

**Affiliations:** ^1^ Institute of Innovation and Applied Research in Chinese Medicine, Hunan University of Chinese Medicine, Changsha, Hunan, China; ^2^ Scientific Research Department, The First Hospital of Hunan University of Chinese Medicine, Changsha, Hunan, China

**Keywords:** natural compounds, mitochondrial dysfunction, target organ damage, hypertension, flavonoids

## Abstract

Hypertension generally causes target organ damage (TOD) in the heart, brain, kidney, and blood vessels. This can result in atherosclerosis, plaque formation, cardiovascular and cerebrovascular events, and renal failure. Recent studies have indicated that mitochondrial dysfunction is crucial in hypertensive target organ damage. Consequently, mitochondria-targeted therapies attract increasing attention. Natural compounds are valuable resources for drug discovery and development. Many studies have demonstrated that natural compounds can ameliorate mitochondrial dysfunction in hypertensive target organ damage. This review examines the contribution of mitochondrial dysfunction to the development of target organ damage in hypertension. Moreover, it summarizes therapeutic strategies based on natural compounds that target mitochondrial dysfunction, which may be beneficial for preventing and treating hypertensive target organ damage.

## 1 Introduction

Over 1.2 billion individuals suffer from hypertension worldwide, and its prevalence is projected to rise (Benjamin et al., 2018). It can damage essential organs, such as the heart, kidney, brain, and blood vessels, resulting in severe complications, such as stroke, heart attack, and kidney failure. These complications are associated with high disability and mortality rates and significant social and economic costs (Zhou et al., 2021). Therefore, preventing and treating hypertensive target organ damage (TOD) are crucial global challenges. Novel therapeutic techniques are necessary to prevent hypertensive TOD and enhance cardiovascular outcomes.

Mitochondria are essential organelles with two membranes that serve as the primary source of cellular energy. They are necessary for cardiovascular function because they generate adenosine triphosphate (ATP), control intracellular calcium ions (Ca^2+^), and metabolize amino acids and fatty acids (Paggio et al., 2019; Abdel-Rahman et al., 2021). Over the past two decades, experimental research has demonstrated that hypertension can cause aberrant mitochondrial function in various tissues, including the heart, brain, kidneys, and blood vessels. In hypertensive TOD, mitochondrial dysfunction is characterized by mitochondrial energy dysregulation, impairment of biogenesis and dynamics, and oxidative damage to DNA, proteins, and lipids in the cell caused by excess generation of reactive oxygen species (ROS). Accordingly, hypertensive TOD pathogenesis and progression depend greatly on mitochondrial function (Shirakabe et al., 2016; Dikalova et al., 2020; Forte et al., 2020).

Several studies have explored mitochondrial dysfunction as a potential therapeutic target (Vaka et al., 2018; Li et al., 2019a). Some anti-hypertensive drugs can reduce mitochondrial damage caused by hypertension, however, therapies that directly target mitochondria may be more effective in reducing hypertensive mitochondrial malfunction or organ damage (de Cavanagh et al., 2006). Natural compounds affecting mitochondrial function have emerged as promising candidates for treating hypertensive TOD. However, there is a lack of comprehensive research regarding the efficacy of natural compounds targeting mitochondrial dysfunction for treating and managing hypertensive TOD. Here, we summarize the mechanism of mitochondrial dysfunction in hypertensive TOD and some recent natural compound-based treatments targeting mitochondrial dysfunction.

## 2 A key role of mitochondria in physiology

Mitochondria are double-membrane organelles between 0.5 and 1 µm in diameter that hold their genetic material (Shpilka et al., 2021). However, their genomes are limited, and most proteins are derived from the nuclear genome (Münch and Harper, 2016). Mitochondria are the powerhouses of cells as they produce the majority of ATP by aerobic cellular respiration. Mitochondria are also involved in various other cellular functions, including cell differentiation, signal transduction, apoptosis, cell proliferation, and cell cycle regulation (Lampert et al., 2019). Therefore, mitochondrial activity is essential for cellular homeostasis and survival.

Mitochondrial dysfunction may be caused by abnormalities in mitochondrial morphology and structure, poor ATP synthesis, excessive ROS production, dysregulated Ca^2+^ handling, dynamics imbalance, and modulating mitochondrial DNA (mtDNA) damage (Zhou et al., 2019; Xue et al., 2020). These modifications can affect cellular energy metabolism, redox balance, and signaling pathways. The pathogenesis and progression of various human diseases have been linked to mitochondrial malfunction. Consequently, mitochondrial targeting is essential for disease treatment.

Mitochondrial dysfunction can result from abnormalities in mitochondrial morphology and structure, impaired ATP synthesis, excessive ROS production, dysregulated Ca^2+^ handling, dynamics imbalance, and mtDNA damage (Zhou et al., 2019; Xue et al., 2020). These alterations can affect cellular energy metabolism, redox balance, and signaling pathways. Mitochondrial malfunction has been associated with the pathogenesis and progression of various human diseases. Therefore, targeting mitochondria is crucial for disease treatment.

## 3 Mitochondrial dysfunction in hypertensive TOD

Hypertension affects mitochondrial adaptations and degrades mitochondrial function, resulting in decreased ATP production, increased oxidative stress, and loss of mitochondrial integrity (Eirin et al., 2014). Changes like these can damage many organs and cause a steady reduction in mitochondrial function. Hypertension harms to the kidneys, brain, heart, and vasculature through mitochondrial dysfunction-related processes.

### 3.1 Heart

Mitochondrial dysfunction is a key factor in the pathogenesis of cardiomyocyte death and cardiac impairment caused by various cardiovascular diseases (Nargesi et al., 2021). Hypertension-induced cardiac hypertrophy, a common complication that leads to heart failure, can be modeled by spontaneously hypertensive rats (SHRs) and pressure overload rats induced by transverse aortic constriction (TAC) (Musumeci et al., 2011). Heart failure is characterized by reduced energy production due to mitochondrial dysfunction, which affects complex I activity, ATP levels, and mitochondrial ROS (mtROS) generation (Shen et al., 1999; Dai et al., 2011a). Cardiomyocytes have substantial energy demands, with mitochondria occupying 30% of their intracellular volume and producing 6–30 kg of ATP daily (Weiss et al., 2005). Heart failure could alters mitochondrial dynamics, shifting toward fission and mitochondrial disintegration (Wai et al., 2015). Mitochondrial malfunction also contributes to diverse forms of myocyte death in heart failure (Li et al., 2019b). Consequently, mitochondria have a significant role in the progression of hypertensive heart disease structurally and functionally.

### 3.2 Vessels

Research has demonstrated that hypertension induces mitochondrial dysfunction in vascular endothelial and smooth muscle cells, elevating reactive oxygen species generation (Dikalova et al., 2010). Dynamics-related protein 1 (DRP1) activation and optic atrophy protein 1 (OPA1) downregulation impairs mitochondrial dynamics in the small mesenteric arteries of SHRs, leading to an increase in mtROS emission, inflammation, and growth factor signaling, which causes medial thickening and luminal constriction (Li et al., 2022a). Vascular mitochondrial dysfunction can stimulate proliferation and phenotypic switching from contractile to proliferative in vascular smooth muscle cells (VSMCs), resulting in vascular remodeling and stiffness (Lu et al., 2021; Ye et al., 2021). Mitochondrial dynamics is essential for VSMCs proliferation (Marsboom et al., 2012). Mitochondrial fission restriction can inhibit VSMCs migration by modifying mitochondrial energy and ROS levels (Wang et al., 2015). Endothelial mitochondria are signaling variables that control endothelial function and ROS generation (Shukla et al., 2020). Mitochondria influence endothelial pathophysiology through Ca^2+^ signaling (Yamamoto et al., 2018), nitric oxide (NO) production (Dedkova et al., 2004), apoptosis and autophagy (Gao et al., 2020), thereby contributing to end-organ damage. Furthermore, numerous studies have demonstrated that hyperacetylation of mitochondrial proteins (SIRT3, SOD2, or CyPD) can impair mitochondrial metabolism and oxidative stress, inducing hypertensive vascular dysfunction (Dikalova et al., 2017; Dikalov and Dikalova, 2019; Dikalova et al., 2020).

### 3.3 Kidney

Hypertension causes glomerular capillary damage and tubulointerstitial inflammation in kidneys. The glomerular filtration rate decreases due to a diminished filtration surface area and nephron count (Lee et al., 2019). The commonly used models are animals with bilateral renal artery stenosis and high salt-induced renal injury model. Hypertension increases the risk of kidney failure by inducing kidney hypoxia (Friederich-Persson et al., 2013). The main contributors to hypoxia in the hypertensive kidney are oxidative stress-mediated excess of ROS and a NO deficiency, which inhibits mitochondrial oxygen utilization (Adler and Huang, 2002). Extensive research indicates that renal medullary hypoxia contributes to the advancement of kidney damage in chronic kidney disease and hypertension (Li et al., 2008). Recent research has demonstrated that mitochondrial malfunction due to a deficiency of apoptosis-inducing factor (AIF) (Coughlan et al., 2016), mitochondrial complex I activity (Forbes et al., 2013), or faulty fatty acid oxidation (Kang et al., 2015) may contribute to the development of chronic kidney disease. Moreover, urine mtDNA is related to renal impairment and dysfunction markers in hypertensive patients, demonstrating mitochondrial damage in kidney impairment in hypertension (Eirin et al., 2016a).

### 3.4 Brain

Hypertension alters cerebral blood flow control and blood-brain barrier permeability. Spontaneously hypertensive stroke rats (SHRSP) exhibit cerebrovascular alterations similar to human disease; they are frequently employed as a model to examine hypertensive brain injury (Fredriksson et al., 1988). Mitochondrial respiratory chain dysfunction and high mtROS levels in SHRSP neurons result in cell death and cognitive impairment (Rubattu et al., 2016). Mitochondria in the brain are essential for sustaining cerebral energy metabolism, antioxidant defense, anti-inflammatory response, and survival and function of neurons. Their dysfunction can precipitate neurodegeneration and increases the risk of cognitive impairment and stroke (Lopez-Campistrous et al., 2008; Sumbalová et al., 2010). Several mitochondrial proteins, including complexes I, II, and IV, adenine dinucleotide translocase (ANT), ATP-sensitive potassium channel (mitoKATP), and mitochondrial permeability transition pore (mPTP), which are involved in energy production, ion transport, and cell death regulation, have been identified as potential neuroprotective targets in hypertensive stroke (Jin et al., 2016). Moreover, brain damage caused by ischemia or trauma can disrupt mitochondrial structure and function in neurons, influencing their neurotransmission and plasticity (Kislin et al., 2017).

## 4 Potential targets for mitochondrial dysfunction

### 4.1 Modulation of mitochondrial dynamics

Mitochondrial dynamics is a process that regulates the size, shape, number, and function of mitochondria in response to the metabolic demands and stress conditions of the cells, which involves four key events: fission, fusion, biogenesis, and mitophagy (Archer, 2013). During fission, DRP1 and mitochondrial fission 1 protein (FIS1) are responsible, while during fusion, mitofusin 1 and 2 (MFN1 and MFN2) and OPA1 are responsible (Archer, 2013). In hypertensive TOD, mitochondrial dynamics are disturbed, contributing to its pathogenesis. Therefore, treatments promoting mitochondrial fusion or preventing fission may reduce hypertensive TOD. Mitochondrial biogenesis, which produces new mitochondria, requires approximately 150 proteins, including transcription factors, enzymes, and receptors. Three key synergistic components are peroxisome proliferator-activated receptor gamma co-activator 1α (PGC-1α), adenylate-activated protein kinase (AMPK), and sirtuin-1 (SIRT1) in mitochondrial biosynthesis (Cantó et al., 2009; Luo et al., 2012). During mitochondrial biogenesis, the cell’s mitochondrial mass and number increase. Mitochondrial autophagy, a quality control program regulated by the PTEN-induced kinase 1 (PINK1)-Parkin pathway, is also essential for hypertensive TOD (Shirakabe et al., 2016) Taken together, the modulation of mitochondrial homeostasis can improve hypertensive TOD.

### 4.2 Modulation of mitochondrial bioenergetics

Mitochondrial bioenergetics involve energy production and conversion in mitochondria by enzymatic and metabolic pathways. Mitochondria in cardiomyocytes and renal tubular cells generate substantial ATP (Suzuki et al., 2016). Mitochondrial respiration involves a series of processes in the inner mitochondrial membrane, including the generation of a proton gradient, movement of electrons by the electron transport chain (ETC.), and phosphorylation of adenosine diphosphate (ADP) to form ATP (Cogliati et al., 2013). Cardiolipin, a critical component of the inner mitochondrial membrane, is necessary for the construction and function of, ETC. Cardiolipin content and composition are reduced in hypertensive animals’ cardiac and renal tissues, indicating impaired mitochondrial bioenergetics (Zachman et al., 2010; Eirin et al., 2016b). Consequently, addressing mitochondrial bioenergetics and increasing ATP production may be crucial in treating hypertensive TOD.

### 4.3 Modulation of mitochondrial oxidative stress

Mitochondria dysfunction is caused by oxidative stress, an imbalance between ROS and intracellular homeostasis. ROS can be generated by xanthine oxidase, NADPH oxidase, and uncoupled NO synthase, among others (Beswick et al., 2001). However, mitochondria are the primary source and target of ROS in cells. Abnormal mitochondrial respiration can impede oxidative phosphorylation and increase ROS generation, resulting in oxidative damage to cellular and mitochondrial proteins, lipids, and DNA, exacerbating mitochondrial dysfunction, and forming a vicious cycle of damage (Satoh et al., 2021).

The pathophysiology of hypertensive TOD is linked to mitochondrial oxidative stress. Several studies have depicted that elevated mtROS production is associated with cardiac hypertrophy (Dai et al., 2011a; Dai et al., 2011b), renal dysfunction (Gu et al., 2015), arterial endothelial dysfunction (Doughan et al., 2008; Dikalov et al., 2014), and cerebrovascular remodeling (Fang et al., 2021) in various hypertension models. Modulating mitochondrial oxidative stress is a potential therapeutic strategy to reduce TOD in hypertension.

### 4.4 Modulation of mitochondrial-mediated apoptosis

Another mechanism connecting mitochondrial dysfunction with hypertensive TOD is mitochondria-mediated apoptosis. Mitochondria are crucial regulators of apoptosis, the process of programmed cell death process that preserves cellular homeostasis and viability. Apoptosis can be triggered by intrinsic or extrinsic pathways (Ivanova et al., 2019). In response to intracellular damage, such as oxidative stress, Ca^2+^ excess, or DNA damage, the intrinsic pathway is initiated by the permeabilization of outer mitochondrial membrane (Cheng et al., 2016). A key step in the intrinsic route, the opening of the mPTP, results in the release of pro-apoptotic proteins, such as cytochrome c and the activation of caspase-9 and caspase-3. These caspases can cleave diverse substrates and induce cell death (Volkova et al., 2011).

It is believed that mitochondria-mediated apoptosis contributes to the pathogenesis of hypertensive TOD. Inhibition of mPTP opening can limit the release of pro-apoptotic proteins and protect against mitochondrial malfunction and cell death. This is possible using mitochondrial antioxidants or cardiolipin-protective agents. Mitochondrial antioxidants such as mitoQ can scavenge mtROS and reduce oxidative damage to mitochondria and cells (Pak et al., 2018; Goh et al., 2019). Cardiolipin-protective agents can maintain cardiolipin content and function and stabilize the inner mitochondrial membrane (Mulligan et al., 2012; Eirin et al., 2016b).

## 5 Natural compounds-based hypertensive TOD targeting mitochondrial dysfunction

Natural compounds are small molecules derived from natural sources, such as plants, animals, fungi, or bacteria. Due to their low side effects and toxicity, they have been a significant source of novel drugs for human diseases. More than 30% of medications approved by the Food and Drug Administration (FDA) contain natural components or their derivatives (Enghiad et al., 2021). In recent years, natural compounds that modulate mitochondrial activity have emerged as a promising area of therapeutic development. Therefore, natural compounds that affect mitochondrial function may have beneficial effects on TOD in hypertensive. This section reviews some natural compounds that modulate mitochondrial activity to treat hypertensive TOD.

### 5.1 Flavonoids

Flavonoids are a large class of low molecular weight compounds widely distributed in foods and medicinal plants. They have various health benefits, including antioxidant, anti-inflammatory, antitumor, and chemopreventive activities (Sayed et al., 2020). Epidemiological evidence suggests that consuming of foods or beverages rich in flavonoids can reduce the incidence of cardiovascular diseases (Mahmoud et al., 2019).

Acacetin (ACT) is a naturally occurring flavonoid isolated from the traditional Chinese medicine *snow lotus*. It has several pharmacological actions, including antimicrobial, anti-inflammatory, anti-proliferative, and anticancer (Singh et al., 2020). Recent *in vivo* and *in vitro* studies have demonstrated that ACT protects against hypertensive TOD. For example, Li et al. reported that ACT protects against vascular endothelial dysfunction in hypertension by activating the AKT/eNOS pathway and targeting mitochondrial mPTP and DRP1/OPA1-dependent regulation of mitochondrial dynamics (Li et al., 2022b). Yuan et al. found that ACT improves cardiac mitochondrial dysfunction by regulating PI3K/AKT signaling pathway-mediated mitochondrial apoptosis, oxidative stress, and mitochondrial fission (MFF, DRP1) and fusion (MFN2) in SHRs with insulin resistance, thus suggesting a beneficial role for ACT in the treatment of mitochondrial dysfunction (Yuan et al., 2022). These results suggested that ACT, which modulates mitochondrial activity, may be a natural remedy for hypertensive TOD.

Naringin (NRG) is one of the most important flavonoids found mainly in the peel and pulp of *Citrus fruits (Rutacea)*. NRG possesses diverse pharmacological actions, including antioxidant, anti-inflammatory, and anti-hypertensive activities (Ikemura et al., 2012). NRG (about 100 mg/kg/day) can reduce systolic blood pressure and improve vascular and ventricular dysfunction in rats fed with a high-carbohydrate, high-fat diet. These beneficial effects of NRG on cardiovascular health are associated with maintaining the structural and functional integrity of mitochondrial preparations. The high respiratory control ratio suggests that NRG can enhance mitochondrial respiratory chain function and lipid metabolism (Alam et al., 2013).

Icariin (ICA) is a flavonol glycoside derived from the Chinese medicinal herb *Epimedium*. It has been used to treat hypertension, Alzheimer’s disease, cerebral ischemia, and depression (Li et al., 2022a). Qian et al. observed that ICA had a protective effect on cardiac remodeling in SHRs and that oral administration of ICA (20 and 40 mg/kg/day) prevented apoptosis of cardiomyocytes and improved left ventricular remodeling and mitochondrial abnormalities. Further mechanistic studies suggested ICA therapy upregulated Bcl-2 and downregulated p53, Bok, Bax and cleaved caspase 3, indicating a possible mechanism of blocking the mitochondrial apoptotic pathway (Qian et al., 2017). Similarly, intragastric administration of Icariside II (ICA II), a form of ICA metabolite, has been demonstrated to reduce blood pressure, promote cardiac function recovery, and improve ventricular remodeling in SHRs. These cardioprotective effects of ICA II were related to downregulating the activation of oxidative stress-associated proteins ASK1, p38 and JNK, inhibiting the expression of p53, Bax and cleaved caspase 3, and upregulation of the expression of Bcl-2 expression in the mitochondrial apoptotic pathway. This process may involve the prevention of mitochondrial apoptosis mediated by the ASK1-JNK/p38 signaling pathway (Wu et al., 2018).

Epigallocatechin-3-gallate (EGCG), a flavonoid found in green tea, has beneficial effects on hypertension and its complications. EGCG (200 mg/kg, i.g. for 12 weeks) protects neuronal against apoptosis by regulating mitochondria-mediated apoptotic pathways, including decreased Bax/Bcl-2, Bak/Bcl-xL, cytochrome C release, caspase-9 activation, and increase SIRT1/PI3K/AKT-related pro-survival pathway. This suggests a therapeutic potential of EGCG for hypertension-induced brain damage (Hsieh et al., 2021). EGCG (50 mg/kg/day, i.p., for 21 days) reduced hypertension-induced ventricular hypertrophy in rats by modulating mtDNA copy number and respiratory chain complexes I, III, and IV (Chen et al., 2009). EGCG may be a promising natural compound for treating hypertensive brain and heart damage by altering mitochondrial function.

Quercetin (QEC) is a prevalent flavonoid found in numerous plants. It has antioxidative properties due to ortho-diphenolic hydroxyl groups at B-rings and double bonds at C-rings (Sekher Pannala et al., 2001). Cui et al. (2017) reported that QEC prevents mitochondrial dysfunction, including increases the mitochondrial potential and ATP production and inhibits phosphate-induced apoptosis and calcification of VSMCs by reducing oxidative stress and mitochondrial fission by downregulating DRP1 expression and phosphorylation. Similarly, recent research has demonstrated that QEC can also improve cardiac function by reducing mitochondrial superoxide and preserving mitochondrial structure *in vivo*. It also attenuated angiotensin II (Ang II) -induced cardiac hypertrophy *in vitro*. Notably, mitochondrial protection and PARP-1 suppression by QEC were partially abolished by SIRT3 knockdown. These findings imply that QEC suppresses cardiac hypertrophy by regulating the SIRT3/PARP-1 pathway to preserve mitochondrial function (Chen et al., 2021a).

Dihydromyricetin (DHY), a flavonoid derived from *Garcinia cambogia,* possesses anticancer, antioxidant, anti-inflammatory, and neuroprotective properties (Zhang et al., 2018a). DHY pretreatment (250 mg/kg/day, i.g, for 4 weeks) could significantly improve cardiac function and reduce cardiac index after TAC-induced hypertrophy. DHY inhibited oxidative stress by decreasing ROS generation and malondialdehyde levels, and increasing total antioxidant capacity and superoxide dismutase activity in the myocardium. Importantly, DHY enhanced the expression and activity of SIRT3, a key regulator of mitochondrial function and oxidative stress resistance, as well as its downstream targets, forkhead-box-protein 3a (FOXO3a) and SOD2, in the myocardium. These findings imply that DHY ameliorates pressure overload-induced myocardial hypertrophy in mice by activating the SIRT3 pathway and decreasing oxidative stress (Chen et al., 2018).

Baicalein (BCL) is a naturally occurring flavonoid found in the roots of *Scutellaria baicalensis* that exhibits several pharmacological activities, including antibacterial, antioxidation, anticancer, and antiviral (Liao et al., 2019). Cai et al. demonstrated that BCL is a promising treatment for cardiac hypertrophy in rats with abdominal aortic constriction (AAC). These benefic effects of BCL were related to modulating the SIRT3/LKB1/AMPK signaling pathway. Further mechanism research indicates that BCL inhibits proteasome degradation and activates the 20S proteasome subunit beta Type 5 (PSMB5) to promote the production of SIRT3 protein, a key mitochondrial deacetylation modifying enzyme that affects mitochondrial structure and function. These results demonstrated that BCL inhibits cardiac hypertrophy by a SIRT3-dependent mechanism, indicating its potential use in treating cardiac hypertrophy and heart failure (Cai et al., 2023).

### 5.2 Phenolics (not including flavonoids)

Phenolics are natural organic compounds with phenolic hydroxyl groups. Polyphenols, a subgroup of phenolics, have garnered popular interest due to their oxidative coupling capacity. By controlling oxidative stress or associated signaling pathways, notably by limiting mitochondrial damage, they can protect against hypertensive TOD (Table 1).

**TABLE 1:** Natural compounds ameliorate hypertensive TOD by regulating mitochondrial dysfunction.

Category	Natural compounds	Target organs	*In vivo* mode (s)	*In vitro* model (s)	Effect	Possible mechanisms/target	References
Flavonoid	Acacetin (ACT)	Vascular	SHRs (ACT 10, 20 mg/kg)	HUVECs treated with Ang II (ACT 3 µM)	mitochondrial dynamics promotion, apoptosis inhibition, ROS reduction, ATP production	AKT/eNOS pathway, mPTP, DRP1/OPA1, CypD	Li et al. (2022a)
		Heart	SHRs fed with fructose (ACT 25, 50 mg/kg)	H9C2 cells stimulated with H_2_O_2_ (ACT 5 µM)	mitochondrial dynamics promotion, ROS reduction, mitochondrial apoptosis inhibition, ATP production	PI3K/AKT pathway, MFF, DRP1, MFN2 Bax, Bcl-2, cytochrome c, NRF2, Keap1	Yuan et al. (2022)
	Naringin (NRG)	Vascular, Heart	High fat-fed rats (NRG 100 mg/kg)	**--**	ROS reduction, improved mitochondrial bioenergetic	Control respiratory ratio	Alam et al. (2013)
	Icariin (ICA)	Heart	SHRs (ICA 20, 40 mg/kg)	H9C2 cells treated with Ang II (ICA 3 µM)	inhibit mitochondrial apoptosis	Bcl-2, p53, Bax, Bok and cleaved caspase 3	Qian et al. (2017)
	Icariside II (ICA II)	Heart	SHRs (ICA II 4, 8, 16 mg/kg)	--	ROS reduction, inhibit mitochondrial apoptosis	Bcl-2, p53, Bax, cleaved-caspase3, ASK1-JNK/p38 pathway	Wu et al. (2018)
	Epigallocatechin-3-gallate (EGCG)	Brain	SHRs (EGCG, 200 mg/kg)	--	inhibit mitochondrial apoptosis	SIRT1/PI3K/AKT, Bax/Bcl-2, Bak/Bcl-xL, Apaf-1, caspase-9 (	Hsieh et al. (2021)
		Heart	TAC-induced ventricular hypertrophy rats (EGCG, 50 mg/kg)	--	mtDNA copy number promotion	anti-oxidant enzymes and MAPK signals	Chen et al. (2009)
	Quercetin (QEC)	Heart	SHRs (QEC 20 mg/kg)	H9C2 cells treated with Ang II (QEC 0.5, 1, and 2 μM)	Reduce mitochondrial superoxide, protect mitochondrial structure	SIRT3/PARP-1 pathway	Chen et al. (2021a)
		Vascular	adenine-induced aortic calcification rats model (QEC 100 mg/kg)	Pi-treated VSMCs (QEC 50–100 μM)	Increasing ATP production, decreasing mitochondrial fission and mitochondria-dependent apoptotic	Caspase-3, DRP1	Cui et al. (2017)
	Dihydromyricetin (DHY)	Heart	TAC-induced C57BL/6 mice (DHY 250 mg/kg)	---	reduced mitochondrial superoxide, ROS reduction	SIRT3, FOXO3a, SOD2	Chen et al. (2018)
	Baicalein (BCL)	Heart	TAC-induced ventricular hypertrophy rats (BCL 12.5, 25, 50 mg/kg)	Ang II-induced neonatal rat cardiomyocytes (BCL 2.5–20 µM)	mitophagy promotion, ROS reduction	SIRT3/LKB1/AMPK	Cai et al. (2023)
Phenolics (Not include Flavonoids)	Resveratrol (RSV)	Heart	dTGR treated with Ang II (RSV 800 mg/kg)	---	mitochondrial biogenesis promotion	PGC-lα, TFAM, NRF1, Cox4, SIRT1	Biala et al. (2010)
			HS-NT (RSV 18 mg/kg)	---	mitochondrial mass and biogenesis preservation, mitochondrial fatty acid oxidation protection	PPARα	Rimbaud et al. (2011)
		Kidney	High fat-fed rats (RSV 50 mg/L in drinking water)	---	mitochondrial function protection	---	Banday and Lokhandwala (2020)
	Curcumin (CCM)	Kidney	L-NAME- induced albino rats (CCM 100 mg/kg)	---	Apoptosis reduction, mtDNA preservation, ROS reduction	AT1R, Bcl-2, caspase-3	Greish et al. (2020)
		Brain, Vascular	SHRSP (CCM 100 mg/kg)	HUVECs stimulated by H_2_O_2_ (CCM 0.01–5 µM)	mtROS reduction	UCP2 signaling	Lan et al. (2018)
	Apocynin (APO)	Vascular	---	Ang II-induced BAECs (APO 0.6 µM)	mtROS reduction, mitochondrial respiration states 3 and 4 recovery	H_2_O_2_	Doughan et al. (2008)
	Punicalagin (PUN)	Heart, Brain	SHRs (pomegranate extract 150 mg/kg)	---	mitochondrial superoxide anion reduction, mitochondrial biogenesis promotion, mitochondrial dynamics and clearance improvement	AMPK- NRF2 pathway	Sun et al. (2016)
	Salvianolic acid D (Sal-D)	Heart	SHRs (Sal-D 1, 3, 10 mg/kg)	Ang II-induced cardiomyocytes (Sal-D 1, 3, 10 µM)	mitochondrial morphology and structure restoration	Ras and PI3K/AKT pathways	Chen et al. (2023)
Alkaloids	Tetrandrine (TET)	Heart	SHRs (TET 50 mg/kg/day)	---	mitochondrial Ca^2+^ reduction	mitochondrial Ca^2+^	Xu and Rao (1995)
	Chelerythrine (CHE)	Vascular	---	Ang II-induced BAECs (CHE 3 µM)	mtROS reduction	H_2_O_2_	Doughan et al. (2008)
		Heart	C57BL/6 mice induced by unilateral nephrectomy and DOCA	Ventricular myocytes (CHE 50 μM)	mtROS reduction	Na^+^ current	Liu et al. (2013)
	Spermidine (SPE)	Heart	C57BL/6J mice (SPE 40 mg/kg)	---	mitophagy and mitochondrial respiration promotion	Atg5	Eisenberg et al. (2016)
	Melatonin (MEL)	Heart	---	Ang II-induced MMECs (MEL 1 mM)	apoptosis reduction, autophagy and mitochondrial membrane potential promotion	Mst1, Beclin1, LC3, and P62	Wang et al. (2023)
Terpenoids	Astaxanthin (ATX)	Vascular	SHRs (ATX 200 mg/kg)	Ang II- induced VSMCs (ATX 10–25 μM)	mtROS reduction, mitochondrial fission reduction, mitophagy and mitochondrial biosynthesis promotion	H_2_O_2_, DRP1, Fis1, PINK, Parkin, mtDNA, TFAM, PGC-1α	Chen et al. (2020)
	Corosolic acid (CRA)	Vascular	ICR male mice and Sprague-Dawley rats (CRA 10, 20 mg/kg)	rat aortic endothelial cells, HUVECs (CRA 0.01, 0.1, 1.0 μM)	mitochondrial fission reduction, mtROS reduction, and mitochondria-dependent apoptosis reduction	AMPK, DRP1 and NOX2, caspase-3	Li et al. (2016)
	Astragaloside IV (ASIV)	Vascular	---	Ang II-induced VSMCs (ASIV 50 μg/mL)	mtROS reduction, Mn-SOD, mitophagy, and mitochondrial biogenesis promotion	PGC-1α, TFAM, Parkin, DRP1	Lu et al. (2015a), Lu et al. (2015b)
Other Compounds	Diallyl trisulfide (DATS)	Vascular	C57BL/6 J mice treated with Ang II (DATS 500 μg/kg)	Ang II-induced VSMCs (DATS 100 μM)	mitochondrial fission reduction, mtROS reduction	ROCK1/DRP1	Lu et al. (2020)
	Trehalose (TRE)	Brain, Kidney	high salt-fed SHRSP	---	mitochondrial mass and mitophagy promotion, mtDNA reduction	TFEB	Forte et al. (2021)

Table 1 Mechanisms of natural compounds and active components in the treatment of hypertensive TOD.

Resveratrol (RSV) is a polyphenol found in *Vaccinium* berries and other plants. It reduces blood pressure by modulating mitochondrial biogenesis signaling. Zhang et al. discovered that RSV promoted the expression of mitochondrial fusion-related MFN2 and OPA1 in a model of chronic ocular hypertension (Zhang et al., 2018b). Moreover, RSV ameliorates Ang II-induced cardiac remodeling in double transgenic rats (dTGR) with human renin and angiotensinogen genes by increasing the expression of mitochondrial biogenesis markers, including PGC-1α, mitochondrial transcription factor A (TFAM), and nuclear respiratory factor (NRF). These results indicate that the beneficial effects of RSV are mediated by a blood pressure-dependent pathway and are associated with an increase in mitochondrial biogenesis (Biala et al., 2010). Similarly, RSV (18 mg/kg/day, for 8 weeks) maintains mitochondrial mass and biogenesis in Dahl salt-sensitive rats with hypertension-induced heart failure via preserving mitochondrial fatty acid oxidation and PPARα expression (Rimbaud et al., 2011). Banday and Lokhandwala (2020) reported that dopamine oxidation in high salt-fed rats impairs lysosomal and mitochondrial function and induces renal inflammation. RSV (50 mg/L in drinking water for 6 weeks) did not affect basal mitochondrial oxidation or respiration but protected mitochondrial function, preserved renal function and reduced hypertension.

Curcumin (CCM), a diarylheptanoid compound derived from *turmeric*, possesses diverse pharmacological properties, including anti-inflammatory, antioxidant, anti-liver fibrosis, anti-tumor, and anti-atherosclerosis (Tapia et al., 2012). Due to its low toxicity and negligible side effects, CCM is one of the world’s most utilized natural food pigments. Several studies have depicted that CCM has a possible protective effect against mitochondrial abnormalities in several disease types (Cox et al., 2022). The beneficial effects of CCM (100 mg/kg/day, gavage, 10 weeks) on NG-nitro-L-arginine methyl ester (L-NAME)-induced hypertensive kidney injury were mediated by its antioxidant capacity and downregulation of AT1R, including reduction of apoptosis and preservation of mtDNA (Greish et al., 2020). Lan et al. (2018) observed that CCM administration (100 mg/kg/day, gavage, for 10 weeks) delayed stroke onset and increased SHRSP survival by decreasing ROS and improving endothelial-dependent carotid artery relaxation. Further mechanism studies demonstrated that the mechanism of CCM reducing oxidative stress and increasing NO production to improve vascular endothelial function to prevent hypertensive stroke was related to the activation of uncoupling protein 2 (UCP2) signal, a physiological regulator of mtROS generation.

Apocynin (APO), a natural organic compound derived from the roots of *Apocynum cannabinum*, is frequently used as an NADPH oxidase inhibitor (Kovacevic et al., 2020). APO possesses anti-hypertensive properties by suppressing the release of superoxide ions and inhibiting the transport of p47phox to the mitochondrial membrane (Babior, 2004). APO also inhibits the rise in mitochondrial H_2_O_2_ generation that Ang II induces in bovine aortic endothelial cells (BAECs). Knocking down the p22phox subunit of NADPH oxidase with small interfering RNA diminished Ang II-induced mtROS production. Moreover, Ang II reduced BAECs’ mitochondrial glutathione levels, disrupted mitochondrial respiration states 3 and 4, and lowered the mitochondrial respiratory control ratio. However, apocynin reversed these effects (Doughan et al., 2008).

Punicalagin (PUN) is a polyphenolic compound isolated from pomegranate (*PunicagranatumL.*) leaves that exhibit antioxidant effects (Shao et al., 2016). Pomegranate fruit and juice have traditionally been employed to treat and promote health. Punicalagin-containing pomegranate extract can reduce blood pressure and prevent heart hypertrophy, probably by increasing mitochondrial function in the paraventricular nucleus of hypertensive rats. This could involve increasing mitochondrial biogenesis, enhancing mitochondrial dynamics and clearance, and decreasing mitochondrial superoxide anion levels. The AMPK- NRF2 pathway is a potential mechanism (Sun et al., 2016).

Salvianolic acid D (Sal-D) is a natural compound isolated from a natural herbal *Salvia miltiorrhiza* Bunge with cardiovascular benefits. Sal-D is an important antioxidant from Danshen and has attracted increasing research interest (Lu et al., 2022). Chen et al. (2023) demonstrated that Sal-D can treat hypertension-induced heart failure (HF) by reducing blood pressure, attenuating cardiac remodeling, and enhancing cardiac function. These beneficial effects on the heart may entail the protection of mitochondria by reducing mitochondrial ultrastructure damage and energy charge depletion. Further investigations revealed that Sal-D could improve heart function in SHRs by concurrently blocking the Ras pathway and activating the PI3K/AKT pathway.

### 5.3 Alkaloids

Alkaloids are nitrogen organic compounds widespread in plants, animals, and microorganisms. Certain alkaloids can regulate mitochondrial function and have favorable effects on cardiovascular diseases.

Tetrandrine (TET) is a bisbenzylisoquinoline alkaloid that is obtained from the root of *Stephania tetrandra S Moore*, a plant that can treat cardiovascular diseases. Previous research reported that hypertensive rats with left ventricular hypertrophy induced by DOCA-salt had greater mitochondrial Ca^2+^ levels than normal rats. However, TET administration (50 mg/kg/day, gastric, for 9 weeks) decreased heart mass mitochondrial Ca^2+^ levels in hypertensive rats, indicating that TET alleviates cardiac pressure overload by regulating intracellular Ca^2+^ of myocardial mitochondria (Xu and Rao, 1995).

Chelerythrine (CHE) is a naturally occurring benzo[c] phenanthridine alkaloid present in many plant species, including *Chelidonium majus*, *Sanguinaria Canadensis*, and *Macleaya cordata* (Tang et al., 2017a). It inhibits proteinase C selectively and has a vasodilatory effect on the vasculature. CHE suppressed the increase in mitochondrial H_2_O_2_ generation caused by Ang II in bovine aortic endothelial cells (BAECs), indicating that CHE decreases the effects of Ang II-induced mtROS (Doughan et al., 2008). Liu et al. (2013) reported that chelerythrine improved hypertension-induced nonischemic cardiomyopathy, which was involved in improving mitochondrial ROS overproduction and restoring Na^+^ current in myocardial tissue and myocytes.

Spermidine (SPE) is a natural polyamine ubiquitous in organisms that plays an important role in cell transcription, growth, and differentiation (Maki et al., 2017). Previous research has demonstrated that supplementation with spermidine can reduce blood pressure and enhance heart function by improving cardiac mitochondrial autophagy (mitophagy) and respiration *in vivo* (Eisenberg et al., 2016). SPE protects against mitochondrial dysfunction in cardiovascular disease, indicating its potential as a therapeutic agent against mitochondrial malfunction.

Melatonin (MEL) is a tryptophan-derived natural substance with multiple therapeutic benefits, including antihypertensive properties (Ding et al., 2018). The importance of employing MEL as a therapeutic tool to target mitochondria was emphasized in a previous review covering the role of melatonin in modulating mitochondrial physiology in hypertensive TOD (Baltatu et al., 2017). Wang et al. (2023) discovered that MEL decreased mammalian STE20-like kinase 1 (Mst1) expression, increased autophagy, and decreased apoptosis in myocardial microvascular endothelial cells (MMECs) under hypertension conditions. MEL also increased the mitochondrial membrane potential and autophagosome formation in MMECs, thereby providing cardioprotection effects.

### 5.4 Terpenoids

Terpenoids are a wide group of natural products with a molecular skeleton composed of isoprene units. They are diverse and abundant in nature, especially in plants. They have various physiological functions, including antimicrobial, antihypertensive, antitumor, anti-inflammatory, antioxidant, and immunomodulatory activities (Chen et al., 2022; Hu et al., 2022; Xu et al., 2022). In addition, the significance of terpenoids in alleviating mitochondrial dysfunction cannot be overlooked.

Astaxanthin (ATX) is a red carotenoid nutrient found in numerous living organisms and commonly consumed by humans through food. Due to its potent ability to scavenge free radicals, ATX possesses antioxidant and anti-inflammatory effects that influence biological and pharmacological processes (Hatabu et al., 2020). In SHRs, ATX has been reported to reduce blood pressure and improve vascular remodeling (Monroy-Ruiz et al., 2011). A mechanism study has revealed that ATX can improve hypertensive vascular remodeling by ameliorating mitochondrial structural damage, inhibiting mitochondrial fission (DRP1), increasing mitophagy (Parkin and PINK1) and mitochondrial biosynthesis (PGC-1α), and decreasing mtROS (Chen et al., 2020).

Corosolic acid (CRA) is a pentacyclic triterpenoid with antioxidative, anti-inflammatory, and antihypertension properties (Yamaguchi et al., 2006). CRA protects vascular endothelial homeostasis by regulating DRP1 phosphorylation (Ser637) in an AMPK-dependent manner to prevent mitochondrial fission and suppress NLRP3 inflammasome activation in endothelial cells (Li et al., 2016).

Astragaloside IV (ASIV) is a cycloalkane-type triterpenoid saponin derived from *Astragalus membranaceus* (Fisch.) Bge., a traditional Chinese medicine widely used for cardiovascular diseases (Jing et al., 2021). ASIV reverses Ang II-induced ROS production, NADPH oxidase, xanthine oxidase activities, ΔΨm reduction, and Mn-SOD activity decrease in rat aortic VSMCs. Moreover, ASIV treatment enhances mRNA expression of TFAM and PGC-1α and protein expression of PGC-1α, Parkin, and DRP1 in aortic VSMCs. These findings indicate that ASIV protects aortic VSMCs by suppressing mitochondrial fission and ROS overproduction and increasing mitochondrial autophagy and biogenesis (Lu et al., 2015a; Lu et al., 2015b).

### 5.5 Other compounds

Diallyl trisulfide (DATS) is a major antioxidant component in garlic and its usefulness in cardiovascular illnesses, particularly hypertension, has been studied (Chen et al., 2021a). Besides its antioxidant activity, DATS may also protect mitochondrial function and exert beneficial effects (Liu et al., 2014). A recent study by Lu et al. (2020) found that DRP1-mediated mitochondrial fission enhanced mtROS generation in primary mouse VSMCs, contributing to Ang II-induced VSMC responses. Additionally, Ang II increased the activity of the protein kinase ROCK1, which controls VSMC phenotypic switching and mitochondrial fission by phosphorylating DRP1. However, DATS abrogated the biological effect of Ang II. In an animal model of Ang II-induced vascular remodeling, DATS also significantly alleviated mitochondrial fission, VSMCs differentiation, and vessel wall thickening, which are regulated by the ROCK1/DRP1 pathway. These findings indicate that DATS attenuates Ang II-induced vascular remodeling by suppressing DRP1-mediated mitochondrial fission in a ROCK1-dependent manner.

Trehalose (TRE) is a non-reducing disaccharide that lower organisms including yeasts and tardigrades synthesize. It possesses antioxidant effects and promotes autophagy (Sarkar et al., 2007; Tang et al., 2017b). In an SHRSP model, TRE attenuated renal damage and stroke incidence. These results were linked to a significant improvement in mitochondrial health, including the restoration of cerebral autophagy/mitophagy and mitochondrial mass. Further mechanisms indicate that these effects are linked to increased nuclear translocation of transcription factor EB (TFEB), a critical regulator of autophagy (Forte et al., 2021). Moreover, TRE is a natural substance without side effects and a perfect medicinal molecule. Therefore, it may be a viable dietary supplement to augment pharmaceutical therapy for stroke patients with hypertension.

In summary, numerous studies have demonstrated that natural compounds can improve mitochondrial function in animal and cellular models of hypertensive TOD. Most of these compounds are flavonoids, polyphenols, terpenoids, and alkaloids originating from plants. Thus, they may have therapeutic potential in regulating mitochondrial diseases associated with hypertensive TOD.

## 6 Conclusion and perspectives

Hypertension is a significant global public health issue and the leading risk factor for mortality and cardiovascular, cerebrovascular, and renal diseases. However, its pathogenesis remains unclear. Mitochondrial dysfunction is a prevalent pathobiological mechanism in many diseases (Rubattu et al., 2014; Kućmierz et al., 2021). Targeting mitochondria with small-molecule drugs may be an effective method for preventing and treating hypertensive TOD. This review covers numerous natural substances, such as QEC, RSV, CCM, and MEL, which affect mitochondrial activity in hypertensive TOD.

**FIGURE 1:**
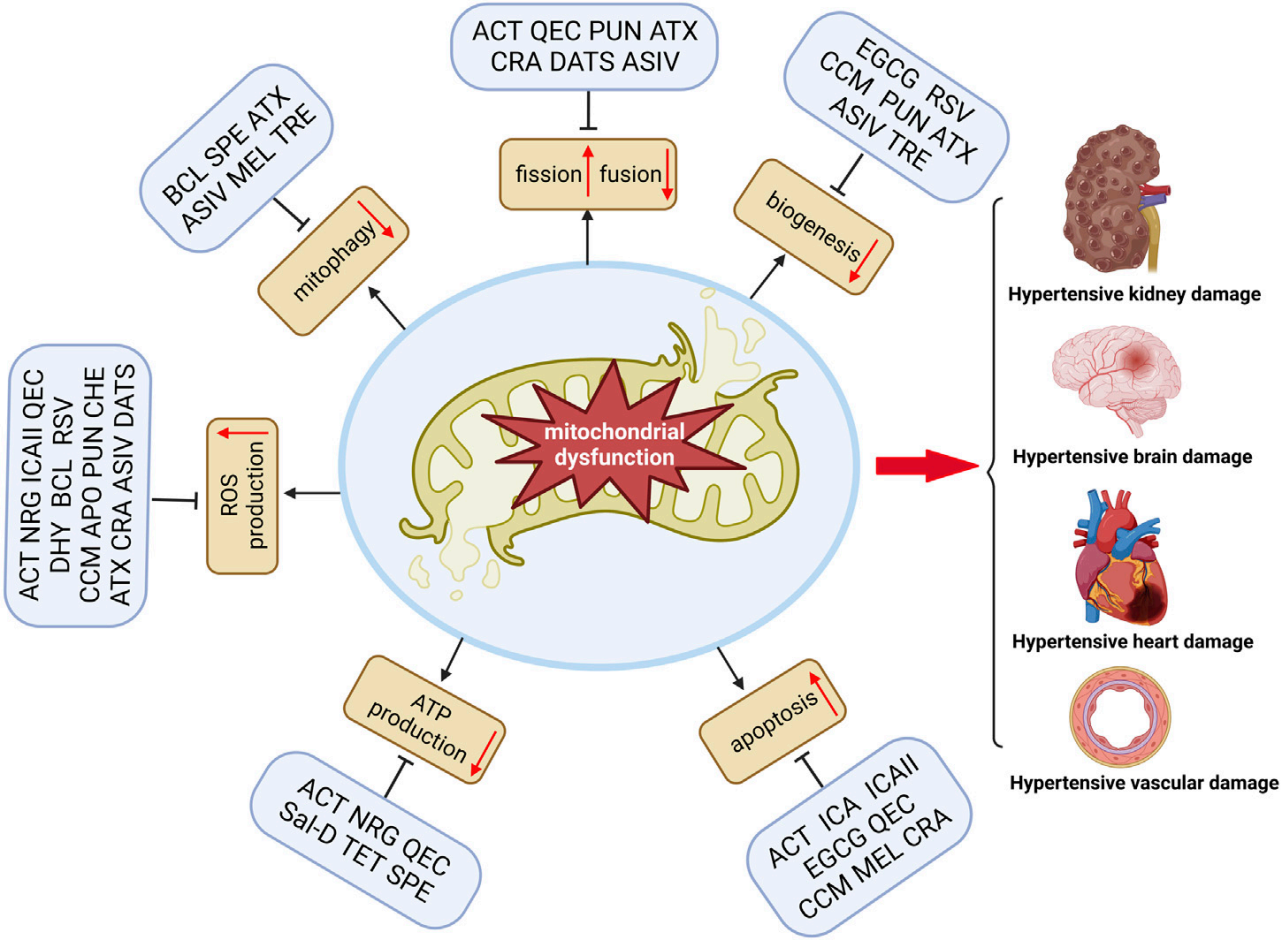
Natural compounds improve TOD in hypertension by modulating mitochondrial dysfunction. Mitochondrial dysfunction includes many aspects, such as reduced mitochondrial biogenesis, reduced ATP production, impaired mitophagy and imbalance fission/fusion, increased ROS production, and increased mitochondrial apoptosis. Natural products, including phenols, terpenoids, alkaloids, and flavonoids, can significantly amend one or several facets associated with mitochondrial dysfunction and thus subsequent amelioration of TOD in hypertension (Figure was created with https://biorender.com).

Most natural products improve mitochondrial function and reduce mitochondrial oxidative stress to prevent TOD in hypertension (Table 1). The balance between antioxidants and oxidants is critical for maintaining mitochondrial homeostasis in hypertensive TOD. Consequently, natural compounds that increase mitochondrial biogenesis and reduce oxidative stress are essential for maintaining mitochondrial function. These compounds have been found to reduce mitochondrial dysfunction and promote cell survival in various hypertensive TOD models. Moreover, as displayed in Figure 1, some compounds, such as RSV, QEC, ACT, and ATX, can modulate multiple aspects of mitochondrial function (biogenesis, dynamics, bioenergetics, or apoptosis) as well as regulate mitochondrial oxidative stress-induced damage. Interestingly, most of these compounds are phenolic compounds, which suggests a protective effect of these natural compounds on hypertensive TOD by targeting mitochondria. This may be due to their antioxidant activity that prevents and attenuates the cytotoxic by-products of cellular respiration and ATP production in damaged mitochondria. Moreover, natural antioxidants can affect mitochondrial biogenesis, dynamics, membrane potential, and apoptosis (Table 1; Figure 1). However, some of the biological activities of phenolic compounds, such as mitochondrial biogenesis and dynamics, are not directly related to their antioxidant activity.

There is experimental evidence that mitochondrial damage contributes to the initiation and progression of hypertensive TOD. Nevertheless, mitochondrial damage’s cause and therapeutic significance in hypertensive TOD remain unclear. Natural compounds that enhance the morphology and function of mitochondria have demonstrated beneficial effects in experimental hypertension. However, their efficacy and safety in alleviating hypertensive TOD need further experimental and clinical validation. Therefore, mitochondria-targeted natural compounds may be effective as adjuvant therapy to standard antihypertensive drugs in treating hypertensive TOD. Future clinical trials in hypertensive patients will require more experimental studies to elucidate their mechanisms of action.
